# Supplementary material for: Baseline Characterization of the Gut Microbiota of Field and Colony Populations of Phlebotomus tobbi and Preliminary Assessment of the Anti-Leishmanial Activity of Cultivable Bacteria
Source: Pathogens. 2026 Jun 23;15(7):658. doi: 10.3390/pathogens15070658 (PMC13416292; doi:10.3390/pathogens15070658)
Supplement: Supplementary file 1 [file pathogens-15-00658-s001.zip › pathogens-4357168-File S1/Heatmap/3.class/heatmap_without_sample_clustering.pdf]

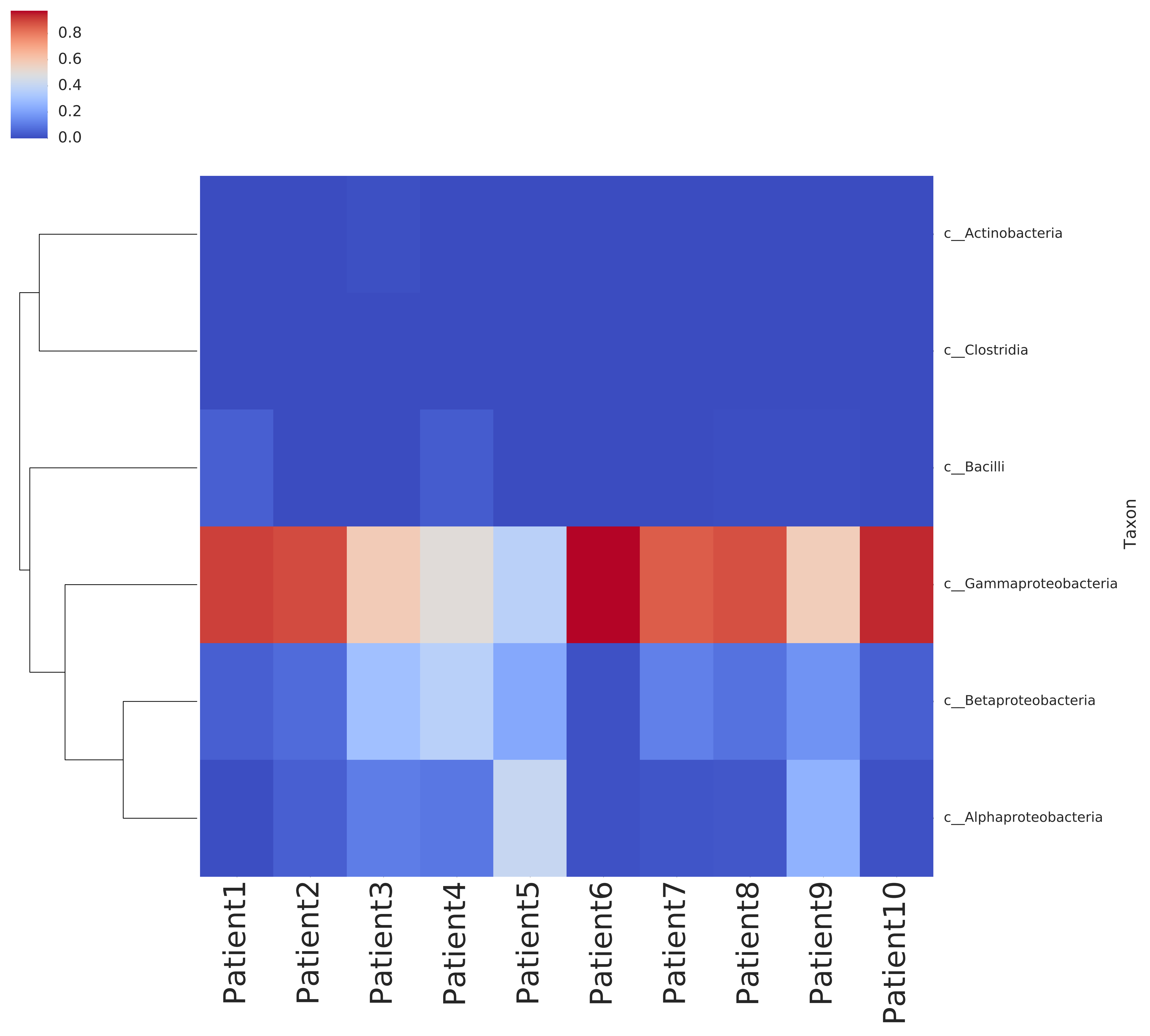

0.8
0.6
0.4
0.2
0.0
c__Actinobacteria
c__Clostridia
c__Bacilli
c__Gammaproteobacteria
c__Betaproteobacteria
c__Alphaproteobacteria
Taxon
Patient1
Patient2
Patient3
Patient4
Patient5
Patient6
Patient7
Patient8
Patient9
Patient10
